# Unleashing the Power of Yes-Associated Protein in Ferroptosis and Drug Resistance in Breast Cancer, with a Special Focus on Therapeutic Strategies

**DOI:** 10.3390/cancers15245728

**Published:** 2023-12-06

**Authors:** RamaRao Malla, Durga Bhavani Kundrapu, Priyamvada Bhamidipati, Ganji Purnachandra Nagaraju, Nethaji Muniraj

**Affiliations:** 1 Cancer Biology Laboratory, Department of Biochemistry and Bioinformatics, GITAM School of Science, GITAM (Deemed to be University), Visakhapatnam 530045, Andhra Pradesh, India; dkundrap@gitam.in (D.B.K.); pkota3@gitam.in (P.B.); 2 Department of Hematology and Oncology, Heersink School of Medicine, University of Alabama, Birmingham, AL 35233, USA; pganji@uab.edu; 3 Center for Cancer and Immunology Research, Children’s National Hospital, 111 Michigan Avenue NW, Washington, DC 20010, USA; nmuniraj@childrensnational.org

**Keywords:** breast cancer, drug resistance, ferroptosis, Hippo-YAP pathway, natural compounds

## Abstract

**Simple Summary:**

The YAP protein is a key component of the Hippo signaling pathway and is frequently overactivated, contributing to drug resistance in cancer. YAP exerts a negative effect on ferroptosis, promoting the survival of cancer cells and resistance to drugs. This investigation explores the potential of natural compounds, either alone or in combination with existing therapies, to target YAP and enhance treatment efficacy. This novel approach holds promise for improving current treatments and advancing the development of new therapies to address drug-resistant breast cancer.

**Abstract:**

The YAP protein is a critical oncogenic mediator within the Hippo signaling pathway and has been implicated in various cancer types. In breast cancer, it frequently becomes activated, thereby contributing to developing drug-resistance mechanisms. Recent studies have underscored the intricate interplay between YAP and ferroptosis within the breast tumor microenvironment. YAP exerts a negative regulatory effect on ferroptosis, promoting cancer cell survival and drug resistance. This review offers a concise summary of the current understanding surrounding the interplay between the YAP pathway, ferroptosis, and drug-resistance mechanisms in both bulk tumor cells and cancer stem cells. We also explore the potential of natural compounds alone or in combination with anticancer therapies for targeting the YAP pathway in treating drug-resistant breast cancer. This approach holds the promise of enhancing the effectiveness of current treatments and paving the way for developing novel therapeutics.

## 1. Introduction

Breast cancer (BC) represents a complex and multifaceted challenge in the realm of clinical oncology. It is characterized by intrinsic heterogeneity, encompassing diverse molecular subtypes such as luminal (luminal A and B) and non-luminal (human epidermal growth factor receptor 2 (HER2)-enriched and basal-like) BCs [1]. The basal-like subtype is characterized by its aggressive features, including a high histological grade and mutations in the TP53 gene. Due to the absence of ER, PR expression, and lack of HER2 enrichment, it is often referred to as triple-negative breast cancer (TNBC). This subtype is strongly associated with the inhibition of BRCA1 function and largely affects younger women. Also, it frequently exhibits overexpression of the EGFR gene [2]. Patients with TNBC exhibit poor survival and an enhanced probability of metastases, particularly to the distant lymph nodes, lungs, and brain [3]. Studies describe the complexity and multifaceted nature of TNBC, underscoring the imperative for a comprehensive understanding of the underlying mechanisms of resistance to established treatment modalities [4]. In recent years, considerable research has been devoted to unraveling the mechanisms underlying chemoresistance in TNBC. 

The emergence of TNBC chemoresistance is increasingly recognized as a complex process that hinges on intricate interactions within the TME. These interactions involve drug efflux mechanisms in bulk tumor cells and cancer stem cells (CSCs), all of which are orchestrated by alterations in multiple signaling pathways [5]. Accumulating evidence underscores that drug resistance in cancer relies on intricate bidirectional interactions between tumor cells and the TME. This interplay involves various components that form a complex three-dimensional network, with a central role attributed to the Hippo-Yes-associated protein (YAP) pathway [6,7]. Furthermore, the TME contributes to drug resistance by modulating ferroptosis, a regulated cell-death mechanism in TNBC [8]. Consequently, comprehending the drug resistance mediated by the Hippo-YAP pathway within the TME, particularly through its impact on ferroptosis, could provide valuable insights for advancing the treatment of drug-resistant TNBC.

Understanding the multifaceted mechanisms of drug resistance arbitrated by the Hippo-YAP pathway, along with its influence on ferroptosis, provides a promising avenue for enhancing treatment strategies in drug-resistant TNBC. This review consolidates the current understanding of the interplay between the Hippo-YAP pathway, ferroptosis, and drug resistance in breast cancer. It also explores the therapeutic implications of targeting these pathways, offering potential improvements in the efficiency of current treatments and the development of novel therapeutics.

## 2. Hippo Signaling Pathway

The Hippo pathway, a key regulator of tissue growth and organ size, was originally reported in *Drosophila melanogaster*. It is conserved evolutionarily from Protista to eukaryotes. Its core consists of both tumor-suppressor and oncogenic proteins. The mammalian Hippo pathway is constituted mainly of tumor suppressor core kinases such as microtubule-associated serine/threonine-protein kinase 1/2 (MST1/2) and large tumor suppressor kinase 1/2 (LATS1/2) along with oncogenic downstream mediators YAP as well as TAZ. In addition, Salvador 1 or WW45, TEA domain family members (TEAD1-4), and Vestigial-like family member 4 are also core components of the Hippo pathway. 

The components of the Hippo pathway are associated with diverse functions, including regulation of transcriptional programs of cell survival and migration as well as self-renewal and differentiation mechanisms. The Hippo pathway is controlled by various intrinsic as well as extrinsic cues like cell–cell contact, stress, cell polarity, mechanical forces, and hormonal factors [9]. Several upstream regulators of core kinases control the components of the Hippo pathway. When the Hippo pathway is activated by upstream activators, MST1/2 is activated and phosphorylates LATS1/2 by complexing with SAV1. Subsequently, phosphorylated LATS1/2 inactivates YAP by phosphorylating serine residue at 127 and TAZ at 66, 89, 117, and 311 and their subsequent binding to TEADs. This phosphorylation promotes the sequestration of YAP/TAZ in the cytosol by enhancing the binding with the 14-3-3 protein. A proteosome-dependent mechanism degrades the phosphorylated YAP/TAZ by interacting with β-TrCP. For example, Merlin (Neurofibromain 2), a tumor suppressor [10]; G-protein coupled receptor [11]; miRNAs [12]; and adherens, tight junctions, and signaling pathways (MAPK, Notch, and Wnt/β-catenin) [13] inhibit the activity of YAP/TAZ by promoting phosphorylation by activating the kinases.

Studies conducted over the past decade consistently reported the loss of Hippo tumor-suppressor activity and hyperactivation of YAP in several cancer types. For example, protein phosphatase 2A, its regulatory subunit STRN3 [14], and its exosomes [15]; posttranslational modification-related kinases (AKT and JNK) [16]; long noncoding RNAs [17]; epigenetic programs [18]; and glucose/GLUT3 inhibit the Hippo pathway and activate YAP by promoting its dephosphorylation [19]. The expression of YAP is induced by WD repeat domain 3 (WDR3) via interaction with GATA binding protein 4 (GATA4) [20]. The inhibition of the Hippo pathway promotes the dephosphorylation of YAP/TAZ and their translocation into the nucleus. They interact with TEAD proteins in the nucleus and trigger target gene expression—notably, the expression of genes controlling survival like CTGF, CRY61, BIRC5, ANKRD1, and AXL. In addition, YAP assists RUNT-related transcription factors (RUNX1 and 2) and SMADs as well as T-box transcription factor 5 (TBX5). Together, this network of genes regulates cell survival and migration as well as self-renewal and differentiation mechanisms.

## 3. YAP Protein and Its Impact on Breast Cancer

YAP expression was reported in different subtypes of BC. An immunohistochemical assessment of YAP expression in BC tissues, along with its correlation with clinicopathological parameters and patient survival, indicates predominant localization within tumor cell nuclei. Notably, YAP expression correlates with PR status and the luminal A subtype. Kaplan–Meier (KM) analyses demonstrate a favorable association of YAP expression with disease-free survival (DFS) and overall survival (OS) in luminal A BC patients. Additionally, a positive association with favorable DFS is observed in patients with invasive ductal carcinoma, luminal B (HER2−), and luminal B (HER2+) BCs [21]. These findings underscore the potential prognostic value of YAP expression in BC, particularly within specific subtypes. In an independent histochemical investigation, higher levels of cytoplasmic YAP and pYAP were observed in HER-2-type BC. Notably, stromal expressions of YAP and pYAP were elevated in luminal B and HER-2-type BCs. Univariate analysis revealed an association between nuclear YAP expression in tumor cells and a shortened OS [22]. These findings emphasize its potential significance as a prognostic indicator, particularly in the context of OS.

Human protein is encoded by the *YAP* gene, which is found on chromosome 11q22 [23]. It was initially reported in chickens as a copartner of yes protein tyrosine kinase. The human form of YAP mediates oncogenesis via interaction with regulators and mediators of oncogenesis through different domains. The SH3 domain of the yes protein tyrosine kinase interacts with YAP in the proline-rich region (PVKQPPPLAP) [24]. A conserved module, namely, the WW domain with two well-preserved and constantly positioned tryptophan residues, was reported in various species of YAP. Such motifs are required for the interaction of YAP with transcription factors having the PPXY motif. In addition, the N-terminal region encompasses a domain for TEAD binding as well as 14-3-3 binding with HXRXXS motifs [25]. The phosphorylation of Ser 127 within the 14-3-3 binding domain develops the 14-3-3 protein binding site and is associated with its cytoplasmic sequestration and inactivation. However, the C-terminus region, having transcriptional activation properties, exhibits resilient transactivation. This region also contains a PDZ-binding motif (FLTWL) that is indispensable for its translocation to the nucleus and binding to the PDZ domain of other regulator proteins such as ZO2 [26]. Additionally, it contains a short coiled–coil motif inside the transcriptional binding domain. 

YAP activation and its subsequent translocation into the nucleus are controlled by two distinct pathways: the canonical Hippo kinase pathway and the noncanonical mechanotransduction pathway [27]. In the Hippo kinase pathway, YAP nuclear accumulation is reduced through its phosphorylation by the Hippo kinases. On the other hand, the mechanotransduction pathway relies on the integrity and force transmission of the actomyosin cytoskeleton. These processes are regulated by a range of oncogenes and tumor-suppressor genes that can either promote or hinder the development of breast cancer.

### 3.1. YAP Mediates Drug Resistance by Inducing Stemness in Breast Cancer Cells

YAP is generally considered a stemness factor due to its role in the overgrowth of organs during embryonic development, including the breast. Its overexpression was confirmed to be critical for the expression of stemness-related genes as well as stemlike signatures and induction of invasiveness in BC patients. Additionally, overexpression of YAP conferred poor outcomes in TNBC patients [28]. Abnormal activity of YAP supports the maintenance of self-renewal by activating TGF-β/BMP pathways, induction of anticancer drug resistance by enhancing the activity of multidrug-resistance-associated (MDR) proteins, and dissemination of cells to facilitate metastasis by activating EMT-related genes [29].

**Figure 1:**
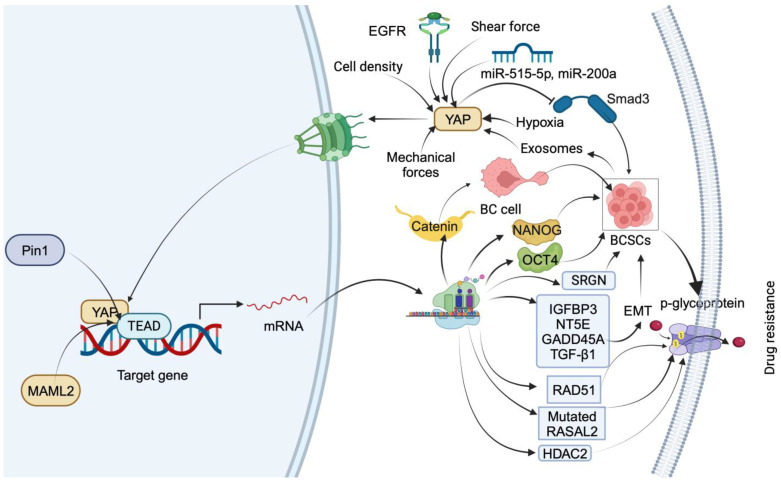
Summary of downstream targets of YAP that mediate drug-resistance mechanisms in TNBC. YAP nuclear translocation is facilitated by EGFR, miR-515-5p, miR-200a, mechanical forces, low shear force, hypoxia, and cell density. In the nucleus, YAP regulates BC cell transdifferentiation into BCSCs by inducing β-catenin expression through interaction with TEAD. YAP induces stemness by transcriptionally upregulating OCT4 and NANOG through interaction with their promoter regions. YAP1 promotes BCSC self-renewal by impeding Smad3 signaling. Exosomes from BC stem cells facilitate drug resistance by upregulating YAP. Pin1 increases drug resistance by stabilizing the YAP complex. YAP1 induces drug resistance and stemness by upregulating IGFBP3, NT5E, GADD45A, TGF-β1, RAD51, and RASAL2 and inducing p-glycoprotein-dependent drug efflux. YAP also triggers drug resistance and stemness by promoting the transcription of HDAC2.

YAP promotes drug resistance through various downstream mediators (Figure 1). The nuclear entry of YAP is controlled by the Hippo pathway via various mechanical and molecular cues. YAP translocates to the nucleus upon activation by receptor tyrosine kinase (Met). The interaction between cytoplasmic EGFR and salt-inducible kinase 2 (SIK2) inhibits the interaction between LATS1 and MST1, thereby facilitating the nuclear translocation of YAP [30]. Furthermore, oncogenic microRNAs, such as miR-515-5p and miR-200a, facilitate nuclear entry by negatively regulating Hippo kinases, while tumor-suppressor microRNAs, including miR-30a and miR-375, impede nuclear entry by positively regulating Hippo kinases [31]. Moreover, mechanical forces like extracellular matrix rigidity and strain trigger YAP nuclear entry by regulating transport across nuclear pores [32]. Low shear force promotes nuclear translocation of YAP via ERK activation [33]. Hypoxia induces the dissociation of 14-3-3ζ from YAP, leading to the promotion of YAP nuclear localization. This process is mediated by ERK2, which directly binds to the D-site of the mitogen-activated protein kinase (MAPK) docking domain in 14-3-3ζ Leu98/100 and phosphorylates 14-3-3ζ at Ser37 [34]. MAML1 and MAML2 act as potent effectors of the Hippo pathway by facilitating YAP/TAZ nuclear localization in a cell-density-dependent manner. Mechanistically, the PPxY-interacting motif within MAML1/2 physically binds to the WW domain of YAP/TAZ, promoting the nuclear retention of YAP/TAZ and subsequent transcriptional activity [35].

In the nucleus, YAP regulates the transdifferentiation of luminal BC cells into BCSCs. YAP induces the expression of β-catenin by interacting with its regulatory elements along with TEAD4. However, the pharmacological intervention of YAP reduces the BCSCs by delaying the transdifferentiation by targeting β-catenin [36]. Another study reported that YAP mediates FOXM1-dependent induction of stemness in TNBC cells. Mechanistically, FOXOM1 promotes nuclear translocation of YAP by diminishing its phosphorylation. YAP transcriptionally upregulates OCT4 and NANOG in the nucleus by interacting with their promoter regions. However, FOXM1 gene silencing reduces the transcriptional activity of YAP by retaining in the cytosol and increasing the proteasomal degradation via phosphorylation [37]. YAP1 promotes the self-renewal of BCSCs by impeding Smad3 signaling. Mechanistically, ectopic expression of YAP enhances the colony formation as well as self-renewal of BCSCs. Also, it inversely regulates Smad3 by inhibiting phosphorylation via targeting TGF-β in BCSCs. However, conditional knockout of YAP1 by infecting cells with adenovirus-Cre reduces the stem cell properties of BCSCs [38]. These studies indicate the role of YAP in the induction of stemness in TNBC cells.

Exosomes derived from BC stem cells facilitate paclitaxel (PTX) resistance by promoting Hippo dysregulation via upregulating YAP [39]. Pin 1, a positive regulator of the Hippo pathway, increases PTX resistance by stabilizing the YAP complex in the nucleus of BC cells [40]. YAP1 also mediates cisplatin resistance in SMARCA2-depleted TNBC cells by upregulating IGFBP3, NT5E, GADD45A, and TGF-β1. However, YAP/TEAD cascade inhibitors, VP and CA3, sensitize SMARCA2 KO TNBC cells by reducing the expression of IGFBP3, NT5E, GADD45A, and TGF-β1 and the EMT pathway [41]. In TNBC cells, cisplatin treatment increases the translocation of YAP into the nucleus by reducing its phosphorylation in an autophagy-dependent manner. However, combining cisplatin and HCQ, an autophagy inhibitor, increases the YAP target gene expression by increasing phosphorylated YAP. Further, silencing of the YAP gene increases the cytotoxicity in TNBC cells by inducing apoptosis. These results indicate that cisplatin-dependent autophagy protects TNBC cells from apoptosis by promoting the nuclear translocation of YAP [42]. In TNBC, YAP/TAZ mediate VEGF- and NRP2-dependent cisplatin resistance by increasing the expression of RAD51, a key mediator of homologous recombination. YAP/TAZ induce the transcription of the RAD51 gene by facilitating the interaction of TEAD4 at the promoter region of RAD51 [43]. The transcriptional profiling of TNBC tumors from a phase 2 clinical trial of platinum chemotherapy predicted that high levels of RASAL2 confer platinum drug resistance in association with transcriptional regulator YAP. Mechanistically, activated YAP transcriptionally enhances the expression of RASAL2 in chemorefractory patient-derived TNBC models [44]. YAP mediates doxorubicin (DOX) resistance in MDA-MB 231 and MDA-MB 468 cells by reducing the uptake of the DOX and inducing p-glycoprotein-dependent drug efflux [45]. YAP mediates serglycin (SRGN) and promotes 5-fluorouracil (5-FU) resistance in BC cells by maintaining stemness. The silencing of YAP sensitizes BC cells to drug resistance induced by the overexpression of SRGN. Further, the YAP/TEAD1 complex promotes the transcription of SRGN. In addition, the YAP/RUNX1 complex promotes the transcription of HDAC2 to trigger 5-FU resistance as well as stemness in BC cells. Mechanistically, YAP and HDAC2 serve as downstream mediators of SRNG-provoked drug resistance in BC patients [46]. These findings could include noteworthy support for YAP as a target for treating drug-resistant BC.

### 3.2. Ferroptosis Factors and Regulation Mechanisms in Breast Cancer

Ferroptosis is a newly discovered form of programmed cell death, characterized by iron-dependent lipid peroxidation and the accumulation of reactive oxygen species (ROS). It is a critical junction point that links cancer-acquired drug resistance and immune evasion. Several mechanisms regulate the sensitivity of cancer cells to ferroptosis by modulating metabolic pathways that control ferroptosis and reshape the TME. These changes lead to the formation of an immunosuppressive environment that promotes tumor growth and progression [47]. 

The mechanism of ferroptosis involves the release of interferon γ (IFNγ) by CD8+ T cells, which leads to the downregulation of two subunits of system Xc-, namely, SLC3A2 and SLC7A11. This, in turn, inhibits cystine uptake by tumor cells, resulting in the depletion of cysteine and glutathione and impaired GPX4 activity, ultimately promoting ferroptosis in tumor cells. However, it is worth noting that the high-fat environment within the TME upregulates the expression of the fatty acid transporter CD36, which increases the sensitivity of CD8+ T cells to ferroptosis and weakens their antitumor effect [48]. 

HLF, a newly discovered oncoprotein of TNBC, has been shown to be regulated by TGF-β1 secreted by TAMs. The mechanism involves HLF activating gamma-glutamyltransferase 1 (GGT1) to enhance resistance to ferroptosis, thereby promoting TNBC cell proliferation, metastasis, and resistance to cisplatin. Additionally, the JAK2/STAT3 axis is induced by IL-6 produced by TNBC cells, leading to the secretion of TGF-β1 by TAMs, thereby creating a positive feedback loop. This interaction between TNBC cells and TAMs promotes the sustained activation of HLF in tumor cells via the IL-6–TGF-β1 axis, ultimately facilitating the development of malignant tumors by promoting ferroptosis resistance through GGT1 [49].

### 3.3. YAP Mediates Resistance to Ferroptosis in Breast Cancer

Emerging evidence suggests that ferroptosis plays a role in the pathogenesis and treatment of TNBC. A study demonstrated the ferroptosis heterogeneity in TNBC [50]. It exhibits diverse phenotypes in terms of ferroptosis-related metabolites and metabolic pathways [51]. A recent investigation revealed that proferroptotic stimuli, including the inhibition of the lipid hydroperoxidase GPX4 and detachment from the extracellular matrix, result in the upregulation of prominin2, a pentaspanin protein associated with the regulation of lipid dynamics, thereby promoting TNBC [52]. Xu et al. reported that elevating GPX4 levels in BC cells leads to an augmentation of tamoxifen (TAM) resistance; conversely, depleting GPX4 in resistant cells decreases TAM resistance [53]. In TNBC cells, miR-324-3p directly targets GPX4. Mechanistically, miR-324-3p binds to the 3′-UTR of GPX4, leading to the downregulation of its expression [54]. One recent study demonstrated that enhanced GPX4 expression, facilitated by m6A modification, promotes BC progression by inhibiting ferroptosis [55].

The intracellular NF2 (Merlin) is activated by E-cadherin-mediated interactions in epithelial cells, suppressing ferroptosis. However, inhibiting this signaling axis allows the YAP to support ferroptosis by increasing the expression of ACSL4 and TFRC. This mechanism sheds light on how intercellular interactions and intracellular NF2-YAP signaling play a crucial role in determining ferroptosis [56]. In BC, mutations in the NF2 gene, leading to loss of function of Merlin, were implicated in the initiation and progression of the disease [57]. Merlin is involved in cell–cell adhesion, cytoskeleton organization, and intracellular signaling pathways, and its dysregulation can contribute to increased cell motility, invasiveness, and resistance to apoptosis [58,59]. The presence of NF2/Merlin mutations in breast cancer has been associated with more aggressive tumor phenotypes and poorer clinical outcomes. BCs harboring NF2 mutations could exhibit increased metastatic potential and resistance to conventional therapies [60]. Another study demonstrated that the suppression of ferroptosis contributes to the progression of TNBC. Mechanistically, the overexpression of progesterone receptor membrane component-1 (PGRMC1) in TNBC results in a reduction in intracellular free iron concentration, consequently inhibiting ferroptosis [61].

Wang et al. recently conducted a study which revealed that YAP plays a crucial role in mediating resistance to ferroptosis. This mechanism involves the downregulation of GPX4 expression. Notably, silencing the YAP gene leads to an increase in ferroptosis, while overexpressing YAP results in ferritinophagy, achieved by reducing mitochondrial ROS and Fe^2+^ levels and lowering the SFXN1 and NCOA4 expression. YAP1 further disrupts the interaction between NCOA4 and FTH1, thus preventing the degradation of ferritin to Fe^2+^. It also reduces ROS production, ultimately suppressing ferroptosis [62]. Lin et al. discovered the significant role of EMT-driven discoidin domain receptor tyrosine kinase 2 (DDR2) upregulation in recurrent breast tumors. This upregulation maintains growth advantage while activating YAP/TAZ-mediated ferroptosis susceptibility [63]. Overall, these findings highlight the importance of YAP in regulating ferroptosis and provide valuable insights into the underlying molecular mechanism involved in the process. 

Ferroptosis has a protective effect on breast cancer (BC) cells [64]. Recent studies have shown that TNBC cells are more sensitive to ferroptosis induction compared to other breast cancer subtypes. This sensitivity is thought to be due to higher iron uptake as well as lipid metabolism in TNBC cells, which are necessary for ferroptosis to occur. In addition, some studies have shown that TNBC tumors have a higher expression of genes implicated in ferroptosis than other BC subtypes. Keiko Miyamoto et al. identified that the expression level of glutamate-cystine transporter (xCT), encoded by the SLC7A11 gene, influences the sensitivity of TNBC to histone deacetylase inhibitors (HDACIs) through the regulation of ferroptosis [65]. SUFU promotes the sensitivity of BC cells to ferroptosis by binding to LATS1, which subsequently suppresses the YAP–ACSL4 axis, leading to a decrease in ferroptosis sensitivity. However, when residues 174–385 are removed from SUFU, this binding interaction between SUFU and LATS1 is disrupted, which results in the inability of SUFU to downregulate the YAP–ACSL4 axis, consequently resulting in the sensitivity of BC cells [66]. Inhibition of YAP confers resistance to ferroptosis; however, YAP overexpression sensitizes BC cells to ferroptosis. A transcriptome study revealed that ferroptosis regulating an E3 ubiquitin ligase (S-phase kinase-associated protein 2; SKP2) is a downstream target gene of YAP. Knockdown of YAP results in the downregulation of SKP2 expression, and both genetic and chemical inhibition of SKP2 provide robust protection against ferroptosis. Moreover, lipid peroxidation during erastin-induced ferroptosis is abolished by the knockdown of YAP or SKP2 [67]. In summary, YAP is a determinant of ferroptosis that regulates SKP2 expression.

A substantial number of studies have established a relationship between YAP and stemness, but limited studies support the association between ferroptosis and stemness in BC. BCSCs initiate the degradation of ferritin within lysosomes in response to cytoplasmic iron depletion, resulting in additional iron accumulation in this cellular organelle. The iron-mediated generation of ROS facilitates lysosomal membrane permeabilization, thereby activating a cell-death pathway consistent with ferroptosis [68]. This mechanistic cascade underscores the significance of iron dynamics and lysosomal processes in driving ferroptotic cell death in BCSCs. These studies reveal that TNBC exhibits diverse ferroptosis phenotypes, with higher sensitivity and distinct molecular features.

### 3.4. YAP in the Control of Metabolic and Oxidative Stress in Breast Cancer

YAP plays a significant role in regulating cellular responses to metabolic and oxidative stress in BC. In conditions of metabolic stress, YAP orchestrates adaptive mechanisms to enhance nutrient utilization and energy production. It promotes glycolysis, the Warburg effect, and lipid metabolism, facilitating cancer cell survival and growth even under nutrient-deprived conditions [69]. Moreover, YAP contributes to redox homeostasis by modulating antioxidant defense systems and ROS levels. It regulates the expression of genes involved in antioxidant pathways, mitigating the detrimental effects of oxidative stress on cancer cells [70]. This dual role of YAP in metabolic adaptation and redox balance positions it as a crucial player in the context of breast cancer progression and survival. The dysregulation of YAP in BC can lead to aberrant metabolic reprogramming and increased susceptibility to oxidative stress, contributing to tumor development and aggressiveness [71,72]. Targeting YAP-associated pathways may hold therapeutic potential in managing the metabolic and oxidative stress responses in BC, presenting avenues for novel treatment strategies.

## 4. Regulation of YAP

The YAP/TAZ complex exhibits both oncogenic and tumor-suppressor functions in a context-dependent manner. Growing evidence suggests that YAP/TAZ interact with various partners to orchestrate oncogenic processes. For instance, the YAP/TAZ complex interacts with TEADs and other proteins during oncogenesis, utilizing their cotranscriptional activation activity to execute distinct functions. Additionally, YAP/TAZ act as transcriptional repressors by interacting with the transcription factor YY1 and the polycomb repressive complex member EZH2 in tumorigenesis. Conversely, YAP/TAZ were identified as playing a tumor-suppressive role by interacting with p73, a homolog of p53, thereby promoting p73-dependent apoptosis and BAX gene expression in response to DNA damage [73].

### 4.1. Tumor-Promoting Role of YAP in Breast Cancer

**Figure 2:**
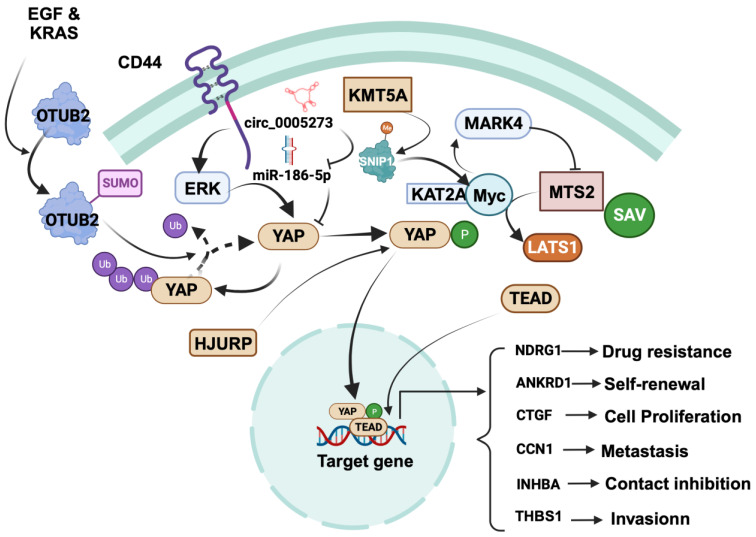
Regulation of cancer-promoting role of YAP in breast cancer. YAP promotes various cellular processes, including proliferation, contact inhibition, invasion, self-renewal, metastasis, and drug resistance. Focusing on specific mechanisms, YAP induces focal adhesion and invasion by driving TEAD-dependent transcription of thrombospondin 1 (THBS1). YAP is positively regulated by CD44, OTUB2, KMT5A, and circRNA hsa_circ_0005273, while it is negatively regulated by SYNPO2 and RICHI1. CD44 activation leads to ERK-mediated YAP phosphorylation, translocating YAP to the nucleus to upregulate ankyrin repeat domain 1 (ANKRD1), connective tissue growth factor (CTGF), cysteine-rich angiogenic inducer 61 (Cyr61/CCN1), and inhibin βA (INHBA). Further regulatory events, such as the SUMOylation of OTUB2 by EGF and KRAS and the stabilization of YAP in the cytosol through deubiquitination, come into play. KMT5A regulates YAP nuclear translocation by catalyzing K301 methylation of SNIP1, releasing histone acetyltransferase KAT2A, and enhancing the c-MYC/KAT2A complex’s recruitment to c-MYC target promoters. This complex activates MARK4, promoting MST2, SAV, and LATS1 phosphorylation, thereby promoting YAP’s nuclear translocation. HJURP affects YAP1 stability and distribution and subsequently binds to the NDRG1 gene promoter, upregulating NDRG1 transcription. Notably, circRNA hsa_circ_0005273 upregulates YAP expression by counteracting miR-186-5p’s tumor-suppressive activity. The figure was created with BioRender.com.

YAP plays a key role in focal adhesion, a key event of invasiveness and metastasis (Figure 2). Mechanistically, YAP promotes focal adhesion and invasion by inducing TEAD-dependent transcription of thrombospondin 1 (THBS1), which in turn activates FAK via phosphorylation at Tyr 397 [74]. TEAD is also essential for YAP-dependent oncogenic transformation as well as EMT [75], negatively regulated by the Hippo pathway [76].

In BC stem cells, an upstream mediator called CD44 is critical in regulating YAP expression. At a mechanistic level, the activation of YAP by CD44 involves the activation of ERK, which phosphorylates YAP. The phosphorylated YAP then moves from the cytosol to the nucleus, where it promotes the expression of genes that are tangled with contact inhibition of BC stem cells, including ankyrin repeat domain 1 (ANKRD1), connective tissue growth factor (CTGF), cysteine-rich angiogenic inducer 61 (Cyr61/CCN1), and inhibin βA (INHBA) [77]. Another factor, OTUB2, promotes stem cell formation and metastasis by stabilizing YAP in MDA-MB 231 TNBC cells via deubiquitination. This occurs through the binding of SUMOylated OTUB2 to YAP through its SUMO-interacting motif. Interestingly, the SUMOylation of OTUB2 is induced by EGF and KRAS [78]. 

Mechanistically, KMT5A regulates the nuclear translocation of YAP by catalyzing the K301 methylation of SNIP1, which serves as a signal to release the histone acetyltransferase KAT2A. This facilitates the interaction between c-MYC and KAT2A, leading to the recruitment of the c-MYC/KAT2A complex to the promoters of c-MYC targets. This complex inhibits the Hippo kinase cascade, thereby enhancing TNBC metastasis by activating the transcription of MARK4. Subsequently, MARK4 modifies the phosphorylation of MST2, SAV, and LATS1 to promote the nuclear translocation of YAP [79].

In TNBC, HJURP modulates cell proliferation and chemotherapy drug resistance via the YAP1/NDRG1-mediated signaling axis. Specifically, HJURP inhibits the ubiquitination of YAP1 protein. This modulation occurs through HJURP’s interaction with YAP1, which regulates YAP1’s stability and cellular distribution. Subsequently, YAP1 binds to the promoter region of the NDRG1 gene, resulting in the upregulation of NDRG1 transcription [80].

In BC cells, the circRNA hsa_circ_0005273 is upregulated and has been shown to promote BC progression through upregulation of YAP protein levels and blocking of the tumor-suppressor activity of miR-186-5p. This regulation occurs through the binding of hsa_circ_0005273 to miR-186-5p, resulting in increased expression of YAP1, leading to enhanced cell proliferation in BC cells. In addition, hsa_circ_0005273 also interacts with other signaling pathways that promote BC tumorigenesis, such as the Wnt/β-catenin, as well as PI3K/Akt/mTOR pathways [81].

### 4.2. Tumor-Suppressor Role of YAP in Breast Cancer

**Figure 3:**
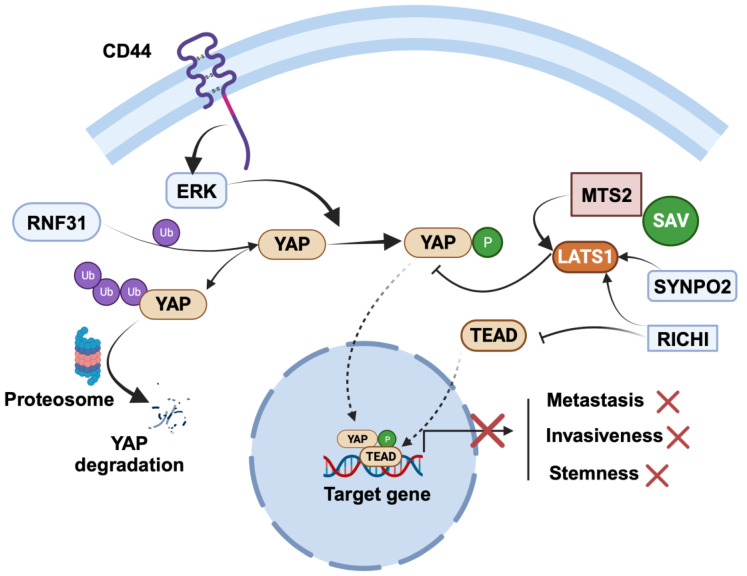
Regulation of cancer-suppressing role of YAP in breast cancer. SYNPO2 and RICHI1 serve as negative regulators of YAP: SYNPO2 stabilizes LATS2, while RICHI1 activates Hippo kinases and inhibits TEAD complexation with YAP. RNF31 interacts with YAP, promoting polyubiquitination and subsequent degradation of YAP. The figure was created with BioRender.com.

YAP is negatively regulated by various mechanisms (Figure 3). YAP is negatively regulated by synaptopodin-2 (SYNPO2) through the stabilization of the LATS2 protein. This essentially inhibits stemness, invasiveness, and metastasis in BC cells via the LATS-mediated inhibition of YAP [82]. Similarly, RICHI1 negatively regulates YAP in BC cells [83]. Mechanistically, the RICHI1-dependent activation of Hippo kinases displaces Amot-p80 from Merlin phosphorylation at S518, resulting in the negative regulation of YAP/TAZ and promotion of transcription of CTGF as well as CYR61 through complexing with TEADs. Overexpression of RICH1 inhibits phosphorylation of MerlinS518 and promotes phosphorylation of LATS1T1079, YAPS127, and TAZS89 while significantly decreasing the total LATS1, YAP, and TAZ protein levels. Studying their interplay may lead to targeted therapies.

RING finger protein 31 (RNF31) is a RING family member with E3 ubiquitin ligases. Studies investigating the role of RNF31 have shown that its silencing leads to increased proliferation and migration of TNBC cells [84]. Clinical data have further reinforced the significance of RNF31, revealing a positive correlation between RNF31 expression and longer relapse-free survival in TNBC patients. Intriguingly, this relationship is inversely associated with YAP protein levels. In-depth molecular biology assays have shed light on the molecular mechanisms underlying RNF31’s tumor-suppressing effects. It has been elucidated that RNF31 has the capacity to interact with YAP protein, facilitating the polyubiquitination and subsequent degradation of YAP, particularly at the K76 sites.

## 5. Targeting YAP Signaling and Drug Resistance in BC

Over the past two decades, a multitude of potential natural compounds and synthetic derivatives have emerged as promising inhibitors of YAP (Table 1). These compounds demonstrate the ability to independently restrain growth and metastasis or sensitize TNBC cells to chemotherapeutic agents through either the inhibition of YAP signaling or the promotion of its degradation. Furthermore, select compounds exhibit the capacity to induce ferroptosis in TNBC cells by modulating YAP expression. 

### 5.1. Selected Natural Compounds Target YAP Signaling and Drug Resistance in BC

Apigenin efficiently reduces the proliferation and migration as well as stemness of TNBC cells [85]. It also reduces the YAP/TAZ activity aside from CTGF and CYR61 expression. Further, it sensitizes TNBC cells to TAZ silencing by interrupting the YAP/TAZ–TEADs association [86]. Luteolin is a type of flavonoid, chemically expressed as 3′,4′,5,7-tetrahydroxyflavone. In TNBC cells, it reduces the activity of YAP/TAZ by triggering the degradation of YAP/TAZ. It also abrogates the EMT by diminishing the expression of vimentin and N-cadherin and enhancing E-cadherin and catenin levels in TNBC cells [87].

The parthenolide derivative called DMOCPTL demonstrates anti-TNBC effects primarily by triggering ferroptosis and apoptosis. It does so by directly binding to and ubiquitinating GPX4. The mechanism uncovered that GPX4 regulates mitochondria-dependent apoptosis by controlling EGR1 in TNBC cells [88]. The prodrug of DMOCPTL efficiently represses the growth of breast tumors and prolongs the lifespan of mice without any noticeable toxicity.

Alantolactone (ALT), a sesquiterpene lactone, was identified as a promising new candidate for treating TNBC by inhibiting the proteins YAP1/TAZ. ALT effectively enhances LATS kinase activities induced by ROS, resulting in increased phosphorylation of YAP1/TAZ proteins. Consequently, the phosphorylated YAP1/TAZ proteins are excluded from the nucleus and subjected to proteasomal degradation, inhibiting tumor growth [89].

In a xenograft tumor model, curcumin (CUR) was demonstrated to have an antitumorigenic effect on BC. It induces ferroptosis via solute carrier family 1 member 5 (SLC1A5) in TNBC cells by increasing the levels of lipid ROS and accumulating malondialdehyde (MDA), an end product of lipid peroxidation and intracellular Fe^2+^ [90]. CUR promotes ferroptotic death and inhibits BC cell viability, which ferrostatin-1, a ferroptosis inhibitor, and deferoxamine, a chelator of iron, can rescue. CUR also significantly increases intracellular Fe^2+^, ROS, lipid peroxides, and MDA and reduces GSH. Moreover, CUR upregulates heme oxygenase-1 (HO-1), a ferroptosis target gene [91]. Another study showed that the novel CUR derivative WZ35 exhibits more potent antitumor effects on BC cells [92]. The mechanism behind this effect involves the generation of ROS and activation of YAP-dependent JNK in BC cells. Subsequently, this activation leads to the dysfunction of mitochondria in BC cells. In contrast, suppression of ROS generation significantly reduces the effects of YAP/JNK activation. WZ35 has shown significant potential as an antitumor agent with improved pharmacokinetic properties. Mechanistically, WZ35 triggers the generation of ROS in TNBC cells, leading to reduced phosphorylation of YAP and increased phosphorylation of JNK. P-JNK triggers apoptosis by phosphorylating the 14-3-3, which facilitates the liberation of proapoptotic proteins such as BAX and FOXO. Additionally, ROS-dependent JNK activation is associated with the dysfunction of mitochondria in BC cells. Activated FOXO transcription factors can reduce N-cadherin, MMP-2, and MMP-9 expression [92]. 

The natural compound resveratrol (RSV), chemically expressed as 3,5,4′-trihydroxy-trans-stilbene, was found to reduce the expression of YAP target genes AREG, CTGF, and CYR61, which are induced by EGF. RSV was observed to attenuate the invasion of TNBC cells by activating LATS1 and the phosphorylation-mediated inactivation of YAP [93]. Overall, these findings suggest that RSV can benefit BC patients by inhibiting BC cell invasion via the suppression of YAP signaling.

Caudatin is a natural steroidal compound that has been shown to reduce the growth of BC cells and the formation of mammospheres. Moreover, it reduces the proportion of CD44^+^/CD24^−^ as well as ALDH^+^ BC cells. It also decreases CD44, Oct4, Sox2, and c-Myc expression. In addition, it was discovered that caudatin promotes the degradation of the glucocorticoid receptor (GR) through ubiquitin-dependent pathways, which inhibits the accumulation of YAP in the nuclei of BCSCs and blocks the transcription of target genes [94]. These findings suggest that the GR/YAP signaling pathway plays a crucial role in BCSC formation and that caudatin may be a chemopreventive agent to target BCSCs.

Rosmarinic acid was found to inhibit the Hippo-YAP/TAZ signaling pathway in MDA-MB 231 cells. The mechanism behind this involves the induction of LATS1/2-driven phosphorylation of YAP, which promotes its cytosol retention and its subsequent degradation. As a result, YAP–TEAD-mediated transcriptional activity is reduced. Additionally, the treatment suppresses YAP/TAZ transcriptional activity by dissociating the YAP/TAZ–TEAD complex [95].

Hydnocarpin is a flavonolignan that exhibits significant effects on TNBC cells. It inhibits TNBC cell proliferation, colony formation, invasion, and EMT [96]. These effects are mediated through the downregulation of CTGF and Cyr61 at both the protein and mRNA levels. Importantly, it reduces total YAP protein levels without affecting YAP mRNA. Overexpression of YAP counteracts the inhibitory actions of hydnocarpin on TNBC cells, confirming the importance of YAP in this context. Furthermore, it enhances YAP degradation via the proteasome pathway and increases YAP ubiquitination, providing mechanistic insights into its anti-TNBC properties.

**Table 1:** Natural compounds and their derivatives targeting YAP-dependent drug resistance and ferroptosis in TNBC.

Natural Compound	Cellular Mechanism	Molecular Mechanism	Ref.
Apigenin	Reduces proliferation of TNBC cells. Inhibits TNBC cell migration. Blocks stemness of TNBC cells.	Reduces the activity of YAP/TAZ. Reduces CTGF and CYR61 expression.	[85]
	Sensitizes TNBC cells to TAZ silencing.	Disrupts protein–protein interaction of YAP/TAZ–TEAD.	[86]
Luteolin	Suppresses EMT in TNBC cells. Reduces migration of TNBC cells.	Triggers the degradation of YAP/TAZ. Diminishes vimentin and N-cadherin expression. Enhances tE-cadherin and catenin expression.	[87]
Parthenolide derivative	Triggers ferroptosis and apoptosis in TNBC cells. Represses breast tumor growth. Prolongs the lifespan of mice without noticeable toxicity.	Binds and ubiquitinates GPX4. Controls GPX4-dependent EGR1 in TNBC cells.	[88]
Alantolactone	Inhibits TNBC growth. Induces ROS in TNBC cells. Sequesters YAP in cytosol by promoting phosphorylation.	Inhibits YAP1/TAZ activity. Enhances LATS kinase activity. Induces proteosomal degradation of YAP.	[89]
Curcumin	Demonstrates antitumorigenic effect on BC. Induces ferroptosis in TNBC cells.	Modulates SLC1A5. Increases ROS. Enhances MDA and intracellular iron.	[90]
	Promotes ferroptotic death. Inhibits BC cell viability.	Increases intracellular Fe^2+^, ROS, lipid peroxides, and MDA. Reduces GSH. Upregulates HO-1.	[91]
Curcumin derivative	Inhibits breast tumor growth. Causes dysfunction of mitochondria.	Suppresses ROS generation. Reduces YAP/JNK activation.	[92]
	Shows potential antitumor activity against TNBC. Induces apoptosis. Triggers ROS generation. Induces mitochondrial dysfunction.	Reduces phosphorylation of YAP. Increases phosphorylation of JNK. Promotes phosphorylation of 14-3-3 and facilitates the liberation of BAX and FOXO. Reduces N-cadherin, MMP-2, and MMP-9 expression by activating FOXO.	[92]
Resveratrol	Attenuates the invasion of TNBC cells.	Reduce EGF-induced YAP-dependent expression of AREG, CTGF, and CYR61. Activates LATS1 and promotes phosphorylation-dependent inactivation of YAP.	[93]
Caudatin	Reduce the growth of BC cells. Suppresses the formation of mammospheres. Reduces the proportion of CD44+/CD24− as well as ALDH+ BC cells. Inhibits self-renewal of BCSCs.	Decreases CD44, Oct4, Sox2, and c-Myc expression. Promotes the degradation of GR through ubiquitin-dependent pathways. Inhibits the accumulation of YAP in the nucleus. Blocks the transcription of YAP target genes.	[94]
Rosmarinic acid	Induces cytotoxicity. Reduces the viability of TNBC cells. Induced cell apoptosis.	Inhibit the Hippo-YAP/TAZ signaling pathway in MDA-MB 231 cells. Induces LATS1/2-driven phosphorylation of YAP. Promotes YAP retention in cytosol and its subsequent degradation. Causes dissociation of YAP/TAZ–TEAD complex. Reduces YAP–TEAD-mediated transcriptional activity.	[95]
Hydnocarpin	Inhibits TNBC cell proliferation, colony formation, invasion, and the EMT.	Downregulates CTGF and Cyr61 at both the protein and mRNA levels. Reduces total YAP protein levels without affecting YAP mRNA. Enhances YAP degradation via the proteasome pathway and increases YAP ubiquitination.	[96]

### 5.2. Selected Inhibitors Target YAP Signaling and Drug Resistance in BC

In BC, verteporfin (VP) was reported to inhibit YAP-dependent tumor growth by disrupting its association with TEAD without light activation in BC (Table 2). VP could block the expression of YAP, pYAP, and TEAD proteins in different types of BC cells, resulting in the disruption of YAP/TEAD interaction. This interference in the YAP-TEAD interaction induces apoptosis by diminishing the expression of survivin and Bcl2 and upregulating levels of cleaved Caspase-9 and cleaved PARP [97]. Jiang et al. demonstrated that VP (4 μM) considerably reduces the invasion as well as migration of TNBC cells by downregulating the expression of YAP and its target genes CYR61 and CTGF [98]. Li et al. demonstrated that VP effectively reduces cell migration while enhancing apoptotic and autophagic responses within PTX-resistant MDA-MB-231 cells, leading to the inhibition of TNBC tumor growth [99]. Additionally, the knockdown of YAP1, a pivotal protein in the Hippo pathway, amplifies the EMT response in PTX-resistant TNBC cells. VP exerts its therapeutic potential against PTX-resistant TNBC by influencing EMT and regulating YAP1, thereby disrupting critical cancer progression pathways. 

Sulaiman et al. reported that nanotherapy using VP and PTX efficiently killed TNBC bulk tumor cells and CSCs in the xenograft model by blocking NF-kB, Wnt, and YAP pathways. Mechanistically, PTX upregulates YAP and its downstream pathways, NF-κB and Wnt. This upregulation of pathways can lead to the enrichment of CSCs in the tumor, which can be detrimental to the prognosis of patients. However, codelivery of VP and PTX using a hybrid nanoparticle-based platform effectively inhibits the YAP-dependent NF-κB and Wnt signaling pathways. As a result, the codelivery approach suppresses the EMT of CSCs in TNBC [100]. In MDA-MB-231 human BC cells, hexasubstituted dipyrrins, which are structurally related to VP, demonstrated significant inhibitory effects on TEAD transcriptional activity as well as on the levels of YAP, TAZ, and the downstream target receptor kinase AXL [101]. Sahli et al. reported that triple-drug nanotherapy using VP, PTX, and combrestatin inhibited the enrichment of stemness in TNBC cells, suppressed the angiogenesis in the zebrafish model, and inhibited PDX tumor growth in an in vivo model [102]. Nanotherapy with VP and PTX inhibits YAP-related pathways, hindering CSC enrichment and enhancing TNBC treatment.

Metformin has been recognized as a potential drug for chemoresistant cancers [103,104]. It has renewed interest as a potential treatment for BC. Metformin treatment exerts an inhibitory effect on BC by inducing apoptosis and G1-phase cell cycle arrest. This treatment also results in downregulating both mRNA and protein expression levels of YAP and TAZ. Additionally, metformin treatment leads to enhanced levels of YAP in the cytosol and a significant decrease in their amounts in the cell nucleus [105]. A study involving 80 BC patients, both TNBC and HER-2+, confirmed that metformin’s effects on BC involve the YAP/TAZ axis [106]. EMT is pivotal in BC, contributing to chemoresistance and metastasis. Metformin inhibits EMT by reducing YAP expression, underscoring its potential as an inhibitor of YAP and a novel BC drug.

Also, metformin has been shown to reduce tumor growth by inducing ferroptosis in an AMPK-independent manner. The mechanism behind this involves an increase in intracellular Fe^2+^ and lipid ROS levels. Metformin particularly targets the protein stability of SLC7A11, a critical regulator of ferroptosis, by inhibiting its UFMylation. Combining metformin with sulfasalazine, a system xc-inhibitor, has a synergistic effect on inducing ferroptosis and inhibiting BC cell proliferation [107].

Aspirin demonstrates a dual mechanism in targeting TNBC [108]. Firstly, it significantly impedes the growth of TNBC cells. Additionally, aspirin exerts its effects by diminishing the expression of YAP and β-catenin. This downregulation is mediated by promoting the activity of the E3 ubiquitin ligase β-TrCP. It effectively eliminates resistance to docetaxel (DTX) and vinorelbine (NVB), two common chemotherapeutic drugs. The cotreatment with aspirin and DTX or NVB synergistically hinders the proliferation of drug-resistant TNBC cells. Furthermore, TNBC patients exhibiting a high expression of YAP level are associated with an elevated risk of relapse and mortality, emphasizing the clinical relevance of a YAP-mediated drug-resistant mechanism.

A supramolecular self-delivery nanomedicine was custom-designed to restore the Hippo pathway’s function [109]. In a highly aggressive TNBC model, the nanomedicine demonstrated remarkable inhibitory effects on tumor growth and metastasis. This nanomedicine undergoes in situ transformation, triggered by elevated levels of GSH and esterase in TNBC cells. It transitions from inert nanospheres to active nanofibers, leading to the controlled release of flufenamic acid, significantly diminishing the transcriptional expression of YAP and related growth-promoting genes. Consequently, the proliferation and metastasis of cancer cells are significantly repressed. Furthermore, restoring the Hippo pathway exhibits remarkable radiosensitization, synergizing with radiotherapy to produce outstanding anticancer activity against TNBC.

**Table 2:** Selected inhibitors target YAP signaling and drug resistance in BC.

Inhibitor	Cellular Mechanism	Molecular Mechanism	Ref.
VP	Inhibits YAP-dependent breast tumor growth. Induces apoptosis.	Disrupts YAP association with TEAD without light activation. Blocks the expression of YAP, p-YAP, and TEAD proteins. Diminishes the expression of survivin and Bcl2. Upregulates cleaved Caspase-9 and cleaved PARP levels.	[97]
	Reduces the invasion as well as migration of TNBC cells.	Downregulates the expression of YAP and its target genes CYR61 and CTGF.	[98]
	Reduces cell migration of PTX-resistant MDA-MB-231 cells. Inhibits EMT in TNBC cells. Enhances apoptotic and autophagic responses. Inhibits TNBC tumor growth.	Downregulates YAP1, thereby disrupting critical cancer progression pathways.	[99]
VP and PTX	Kills TNBC bulk tumor cells and CSCs in the xenograft model. Suppresses the EMT of BCSCs.	Blocks YAP-dependent NF-kB and Wnt pathways.	[100]
Hexasubstituted dipyrrins	Induces cytotoxicity in MDA-MB 231 cells.	Demonstrates inhibitory effects on TEAD transcriptional activity. Reduces the levels of YAP, TAZ, and the downstream target receptor kinase AXL.	[101]
VP, PTX, and combrestatin	Inhibits the enrichment of stemness in TNBC cells. Suppresses the angiogenesis in the zebrafish model. Inhibits PDX tumor growth in in vivo model.	Inhibits the upregulated Hippo/YAP signaling. Inhibits YAP target gene expression.	[102]
Metformin	Shows an inhibitory effect on BC. Induces apoptosis and G1 phase cell cycle arrest.	Downregulates both mRNA and protein expression levels of YAP and TAZ. Enhances YAP levels in the cytosol and decreases them in the cell nucleus.	[105]
	Inhibits EMT in TNBC cells. Reduces drug resistance in TNBC cells.	Reduces YAP expression. Targets YAP/TAZ axis.	[106]
	Reduces tumor growth. Induces ferroptosis in an AMPK-independent manner. Combination with sulfasalazine synergistically induces ferroptosis and inhibits BC cell proliferation.	Increases intracellular Fe^2+^ and lipid ROS levels. Targets the protein stability of SLC7A11. Inhibits SLC7A11 UFMylation.	[107]
Aspirin	Impedes the growth of TNBC cells. Eliminates resistance to DTX and NVB. Synergistically hinders the proliferation of drug-resistant TNBC cells.	Diminishes the expression of YAP and β-catenin. Promotes the activity of the E3 ubiquitin ligase β-TrCP.	[108]
Flufenamic acid	Demonstrates remarkable inhibitory effects on tumor growth and metastasis. Exhibits remarkable radiosensitization in TNBC cells.	Elevates GSH and esterase levels in TNBC cells. Diminishes the transcriptional expression of YAP and related growth-promoting genes.	[109]

## 6. Conclusions and Future Directions

YAP protein plays a pivotal role in mediating the oncogenic effects of the Hippo signaling pathway and contributing to the development of drug-resistance mechanisms in different BC subtypes. In BC, the localization and activity of YAP are controlled by distinct mechanisms. YAP mediates drug resistance in the more challenging BC subtype TNBC by inducing self-renewal and differentiation mechanisms as well as activating multidrug-resistance-associated proteins. YAP also mediates resistance to ferroptosis, a key regulatory cell-death mechanism in TNBC, by modulating critical mediators of ferroptosis. This review synthesized the existing knowledge of and shed light on the interplay between the complex network of molecular interactions that drive resistance to therapy. The breast TME has been identified as a key battleground where the Hippo-YAP pathway and ferroptosis intersect. This intersection, which promotes cancer cell survival and drug resistance, presents a formidable challenge in treating BC. Understanding the molecular crosstalk between these pathways is essential for developing more effective therapeutic strategies.

The potential therapeutic implications of targeting the Hippo-YAP pathway in drug resistance mechanisms are significant. Targeting these pathways could improve the efficacy of current treatments and provide new avenues for developing novel therapeutics. The targeting of the YAP pathway could induce ferroptosis in chemotherapy and prevent the development of drug resistance in BC. Similarly, activation of ferroptosis could induce cancer cell death and enhance the effectiveness of chemotherapy. Also, targeting CSCs could also enhance the effectiveness of chemotherapy by sensitization. These approaches, either alone or in combination with natural compounds that target YAP, could improve the outcomes of breast cancer treatment and provide new opportunities for the development of more effective therapies.

## Data Availability

The data presented in this study are available in this article.

## Abbreviations

ALT	Alantolactone
BC	Breast cancer
CSCs	Cancer stem cells
CUR	Curcumin
DOX	Doxorubicin
DTX	Docetaxel
5-FU	5-fluorouracil
GATA4	GATA binding protein 4
GR	Glucocorticoid receptor
HER2	Human epidermal growth factor receptor 2
HO-1	Heme oxygenase-1
LATS1/2	Large tumor suppressor kinase 1/2
MDA	Malondialdehyde
MDR	Multidrug-resistance associated
NVB	Vinorelbine
PTX	Paclitaxel
RNF31	RING finger protein 31
RSV	Resveratrol
SKP2	S-phase kinase-associated protein 2
SLC1A5	Solute carrier family 1 member 5
SRGN	Serglycin
SYNPO2	Synaptopodin-2
TBX5	T-box transcription factor 5
TEAD1-4	TEA domain family members
THBS1	Thrombospondin 1
TNBC	Triple-negative breast cancer
TME	Tumor microenvironment
VP	Verteporfin
WDR3	WD repeat domain 3
YAP	Hippo/yes-associated protein

## References

[1] Prat A., Pineda E., Adamo B., Galván P., Fernández A., Gaba L., Díez M., Viladot M., Arance A., Muñoz M. (2015). Clinical implications of the intrinsic molecular subtypes of breast cancer. Breast.

[2] Kobayashi S. (2008). Basal-like subtype of breast cancer: A review of its unique characteristics and their clinical significance. Breast Cancer.

[3] Li X., Yang J., Peng L., Sahin A.A., Huo L., Ward K.C., O’Regan R., Torres M.A., Meisel J.L. (2017). Triple-negative breast cancer has worse overall survival and cause-specific survival than non-triple-negative breast cancer. Breast Cancer Res. Treat..

[4] Bai X., Ni J., Beretov J., Graham P., Li Y. (2021). Triple-negative breast cancer therapeutic resistance: Where is the Achilles’ heel?. Cancer Lett..

[5] Nedeljković M., Damjanović A. (2019). Mechanisms of Chemotherapy Resistance in Triple-Negative Breast Cancer-How We Can Rise to the Challenge. Cells.

[6] Yang D., Zhang N., Li M., Hong T., Meng W., Ouyang T. (2021). The Hippo Signaling Pathway: The Trader of Tumor Microenvironment. Front. Oncol..

[7] Wang Z., Wang F., Ding X.Y., Li T.E., Wang H.Y., Gao Y.H., Wang W.J., Liu Y.F., Chen X.S., Shen K.W. (2022). Hippo/YAP signaling choreographs the tumor immune microenvironment to promote triple negative breast cancer progression via TAZ/IL-34 axis. Cancer Lett..

[8] Xu N., Li B., Liu Y., Yang C., Tang S., Cho W.C., Huang Z. (2022). Ferroptosis and triple-negative breast cancer: Potential therapeutic targets. Front. Oncol..

[9] Ma S., Meng Z., Chen R., Guan K.L. (2019). The Hippo Pathway: Biology and Pathophysiology. Annu. Rev. Biochem..

[10] Calses P.C., Crawford J.J., Lill J.R., Dey A. (2019). Hippo Pathway in Cancer: Aberrant Regulation and Therapeutic Opportunities. Trends Cancer.

[11] Luo J., Yu F.X. (2019). GPCR-Hippo Signaling in Cancer. Cells.

[12] Samji P., Rajendran M.K., Warrier V.P., Ganesh A., Devarajan K. (2021). Regulation of Hippo signaling pathway in cancer: A MicroRNA perspective. Cell Signal..

[13] Ouyang T., Meng W., Li M., Hong T., Zhang N. (2020). Recent Advances of the Hippo/YAP Signaling Pathway in Brain Development and Glioma. Cell Mol. Neurobiol..

[14] Tang Y., Fang G., Guo F., Zhang H., Chen X., An L., Chen M., Zhou L., Wang W., Ye T. (2020). Selective Inhibition of STRN3-Containing PP2A Phosphatase Restores Hippo Tumor-Suppressor Activity in Gastric Cancer. Cancer Cell.

[15] Wang S., Su X., Xu M., Xiao X., Li X., Li H., Keating A., Zhao R.C. (2019). Exosomes secreted by mesenchymal stromal/stem cell-derived adipocytes promote breast cancer cell growth via activation of Hippo signaling pathway. Stem Cell Res. Ther..

[16] Yan F., Qian M., He Q., Zhu H., Yang B. (2020). The posttranslational modifications of Hippo-YAP pathway in cancer. Biochim. Biophys. Acta-Gen. Subj..

[17] Zhang S., Liang S., Wu D., Guo H., Ma K., Liu L. (2021). LncRNA coordinates Hippo and mTORC1 pathway activation in cancer. Cell Death Dis..

[18] Jang M., An J., Oh S.W., Lim J.Y., Kim J., Choi J.K., Cheong J.H., Kim P. (2021). Matrix stiffness epigenetically regulates the oncogenic activation of the Yes-associated protein in gastric cancer. Nat. Biomed. Eng..

[19] Wang W., Xiao Z.D., Li X., Aziz K.E., Gan B., Johnson R.L., Chen J. (2015). AMPK modulates Hippo pathway activity to regulate energy homeostasis. Nat. Cell Biol..

[20] Su W., Zhu S., Chen K., Yang H., Tian M., Fu Q., Shi G., Feng S., Ren D., Jin X. (2021). Overexpressed WDR3 induces the activation of Hippo pathway by interacting with GATA4 in pancreatic cancer. J. Exp. Clin. Cancer Res..

[21] Cao L., Sun P.L., Yao M., Jia M., Gao H. (2017). Expression of YES-associated protein (YAP) and its clinical significance in breast cancer tissues. Hum. Pathol..

[22] Kim S.K., Jung W.H., Koo J.S. (2014). Yes-associated protein (YAP) is differentially expressed in tumor and stroma according to the molecular subtype of breast cancer. Int. J. Clin. Exp. Pathol..

[23] Abylkassov R., Xie Y. (2016). Role of Yes-associated protein in cancer: An update (Review). Oncol. Lett..

[24] Yu Y., Su X., Qin Q., Hou Y., Zhang X., Zhang H., Jia M., Chen Y. (2020). Yes-associated protein and transcriptional coactivator with PDZ-binding motif as new targets in cardiovascular diseases. Pharmacol. Res..

[25] Zhao W., Wang M., Cai M., Zhang C., Qiu Y., Wang X., Zhang T., Zhou H., Wang J., Zhao W. (2021). Transcriptional co-activators YAP/TAZ: Potential therapeutic targets for metastatic breast cancer. Biomed. Pharmacother..

[26] Sudol M., Shields D.C., Farooq A. (2012). Structures of YAP protein domains reveal promising targets for development of new cancer drugs. Semin. Cell Dev. Biol..

[27] Morciano G., Vezzani B., Missiroli S., Boncompagni C., Pinton P., Giorgi C. (2021). An Updated Understanding of the Role of YAP in Driving Oncogenic Responses. Cancers.

[28] Yang C.E., Lee W.Y., Cheng H.W., Chung C.H., Mi F.L., Lin C.W. (2019). The antipsychotic chlorpromazine suppresses YAP signaling, stemness properties, and drug resistance in breast cancer cells. Chem. Biol. Interact..

[29] Maugeri-Saccà M., De Maria R. (2016). Hippo pathway and breast cancer stem cells. Crit. Rev. Oncol. Hematol..

[30] Rong X., Liang Y., Han Q., Zhao Y., Jiang G., Zhang X., Lin X., Liu Y., Zhang Y., Han X. (2019). Molecular Mechanisms of Tyrosine Kinase Inhibitor Resistance Induced by Membranous/Cytoplasmic/Nuclear Translocation of Epidermal Growth Factor Receptor. J. Thorac. Oncol..

[31] Sadri F., Hosseini S.F., Rezaei Z., Fereidouni M. (2024). Hippo-YAP/TAZ signaling in breast cancer: Reciprocal regulation of microRNAs and implications in precision medicine. Genes Dis..

[32] Elosegui-Artola A., Andreu I., Beedle A.E.M., Lezamiz A., Uroz M., Kosmalska A.J., Oria R., Kechagia J.Z., Rico-Lastres P., Le Roux A.-L. (2017). Force Triggers YAP Nuclear Entry by Regulating Transport across Nuclear Pores. Cell.

[33] Qin X., Li J., Sun J., Liu L., Chen D., Liu Y. (2019). Low shear stress induces ERK nuclear localization and YAP activation to control the proliferation of breast cancer cells. Biochem. Biophys. Res. Commun..

[34] Jia Y., Li H.-Y., Wang J., Wang Y., Zhang P., Ma N., Mo S.-J. (2019). Phosphorylation of 14-3-3ζ links YAP transcriptional activation to hypoxic glycolysis for tumorigenesis. Oncogenesis.

[35] Kim J., Kwon H., Shin Y.K., Song G., Lee T., Kim Y., Jeong W., Lee U., Zhang X., Nam G. (2020). MAML1/2 promote YAP/TAZ nuclear localization and tumorigenesis. Proc. Natl. Acad. Sci. USA.

[36] Quinn H.M., Vogel R., Popp O., Mertins P., Lan L., Messerschmidt C., Landshammer A., Lisek K., Château-Joubert S., Marangoni E. (2021). YAP and β-Catenin Cooperate to Drive Oncogenesis in Basal Breast Cancer. Cancer Res..

[37] Sun H.L., Men J.R., Liu H.Y., Liu M.Y., Zhang H.S. (2020). FOXM1 facilitates breast cancer cell stemness and migration in YAP1-dependent manner. Arch. Biochem. Biophys..

[38] Sun J.G., Chen X.W., Zhang L.P., Wang J., Diehn M. (2016). Yap1 promotes the survival and self-renewal of breast tumor initiating cells via inhibiting Smad3 signaling. Oncotarget.

[39] Guo Z., Guo A., Zhou C. (2021). Breast Cancer Stem Cell-Derived ANXA6-Containing Exosomes Sustain Paclitaxel Resistance and Cancer Aggressiveness in Breast Cancer. Front. Cell Dev. Biol..

[40] Khanal P., Yeung B., Zhao Y., Yang X. (2019). Identification of Prolyl isomerase Pin1 as a novel positive regulator of YAP/TAZ in breast cancer cells. Sci. Rep..

[41] Kim J., Jang G., Sim S.H., Park I.H., Kim K., Park C. (2021). SMARCA4 Depletion Induces Cisplatin Resistance by Activating YAP1-Mediated Epithelial-to-Mesenchymal Transition in Triple-Negative Breast Cancer. Cancers.

[42] Jiang Y., Ji F., Liu Y., He M., Zhang Z., Yang J., Wang N., Zhong C., Jin Q., Ye X. (2017). Cisplatin-induced autophagy protects breast cancer cells from apoptosis by regulating yes-associated protein. Oncol. Rep..

[43] Elaimy A.L., Amante J.J., Zhu L.J., Wang M., Walmsley C.S., FitzGerald T.J., Goel H.L., Mercurio A.M. (2019). The VEGF receptor neuropilin 2 promotes homologous recombination by stimulating YAP/TAZ-mediated Rad51 expression. Proc. Natl. Acad. Sci. USA.

[44] Koh S.B., Ross K., Isakoff S.J., Melkonjan N., He L., Matissek K.J., Schultz A., Mayer E.L., Traina T.A., Carey L.A. (2021). RASAL2 Confers Collateral MEK/EGFR Dependency in Chemoresistant Triple-Negative Breast Cancer. Clin. Cancer Res..

[45] Qin X., Lv X., Li P., Yang R., Xia Q., Chen Y., Peng Y., Li L., Li S., Li T. (2020). Matrix stiffness modulates ILK-mediated YAP activation to control the drug resistance of breast cancer cells. Biochim. Biophys. Acta Mol. Basis Dis..

[46] Zhang Z., Qiu N., Yin J., Zhang J., Liu H., Guo W., Liu M., Liu T., Chen D., Luo K. (2020). SRGN crosstalks with YAP to maintain chemoresistance and stemness in breast cancer cells by modulating HDAC2 expression. Theranostics.

[47] Friedmann Angeli J.P., Krysko D.V., Conrad M. (2019). Ferroptosis at the crossroads of cancer-acquired drug resistance and immune evasion. Nat. Rev. Cancer.

[48] Yun T., Liu Z., Wang J., Wang R., Zhu L., Zhu Z., Wang X. (2022). Microenvironment immune response induced by tumor ferroptosis-the application of nanomedicine. Front. Oncol..

[49] Li H., Yang P., Wang J., Zhang J., Ma Q., Jiang Y., Wu Y., Han T., Xiang D. (2022). HLF regulates ferroptosis, development and chemoresistance of triple-negative breast cancer by activating tumor cell-macrophage crosstalk. J. Hematol. Oncol..

[50] Jiang L., Gao X.M., Cao J. (2023). The Achilles heel of TNBCs: Ferroptosis heterogeneity. Cell Metab..

[51] Yang F., Xiao Y., Ding J.H., Jin X., Ma D., Li D.Q., Shi J.X., Huang W., Wang Y.P., Jiang Y.Z. (2023). Ferroptosis heterogeneity in triple-negative breast cancer reveals an innovative immunotherapy combination strategy. Cell Metab..

[52] Brown C.W., Amante J.J., Chhoy P., Elaimy A.L., Liu H., Zhu L.J., Baer C.E., Dixon S.J., Mercurio A.M. (2019). Prominin2 Drives Ferroptosis Resistance by Stimulating Iron Export. Dev. Cell.

[53] Xu Z., Wang X., Sun W., Xu F., Kou H., Hu W., Zhang Y., Jiang Q., Tang J., Xu Y. (2023). RelB-activated GPX4 inhibits ferroptosis and confers tamoxifen resistance in breast cancer. Redox Biol..

[54] Hou Y., Cai S., Yu S., Lin H. (2021). Metformin induces ferroptosis by targeting miR-324-3p/GPX4 axis in breast cancer. Acta Biochim. Biophys. Sin..

[55] Ye F., Wu J., Zhang F. (2023). METTL16 epigenetically enhances GPX4 expression via m6A modification to promote breast cancer progression by inhibiting ferroptosis. Biochem. Biophys. Res. Commun..

[56] Wu J., Minikes A.M., Gao M., Bian H., Li Y., Stockwell B.R., Chen Z.N., Jiang X. (2019). Intercellular interaction dictates cancer cell ferroptosis via NF2-YAP signalling. Nature.

[57] Morrow K.A., Das S., Metge B.J., Ye K., Mulekar M.S., Tucker J.A., Samant R.S., Shevde L.A. (2011). Loss of tumor suppressor Merlin in advanced breast cancer is due to post-translational regulation. J. Biol. Chem..

[58] White S.M., Avantaggiati M.L., Nemazanyy I., Di Poto C., Yang Y., Pende M., Gibney G.T., Ressom H.W., Field J., Atkins M.B. (2019). YAP/TAZ Inhibition Induces Metabolic and Signaling Rewiring Resulting in Targetable Vulnerabilities in NF2-Deficient Tumor Cells. Dev. Cell.

[59] Petrilli A.M., Fernández-Valle C. (2016). Role of Merlin/NF2 inactivation in tumor biology. Oncogene.

[60] Mota M., Metge B.J., Hinshaw D.C., Alsheikh H.A., Chen D., Samant R.S., Shevde L.A. (2021). Merlin deficiency alters the redox management program in breast cancer. Mol. Oncol..

[61] Zhao Y., Ruan X., Cheng J., Xu X., Gu M., Mueck A.O. (2023). PGRMC1 promotes triple-negative breast cancer cell growth via suppressing ferroptosis. Climacteric.

[62] Wang J., Zhu Q., Li R., Zhang J., Ye X., Li X. (2022). YAP1 protects against septic liver injury via ferroptosis resistance. Cell Biosci..

[63] Lin C.C., Yang W.H., Lin Y.T., Tang X., Chen P.H., Ding C.C., Qu D.C., Alvarez J.V., Chi J.T. (2021). DDR2 upregulation confers ferroptosis susceptibility of recurrent breast tumors through the Hippo pathway. Oncogene.

[64] Wu M., Zhang X., Zhang W., Chiou Y.S., Qian W., Liu X., Zhang M., Yan H., Li S., Li T. (2022). Cancer stem cell regulated phenotypic plasticity protects metastasized cancer cells from ferroptosis. Nat. Commun..

[65] Miyamoto K., Watanabe M., Boku S., Sukeno M., Morita M., Kondo H., Sakaguchi K., Taguchi T., Sakai T. (2020). xCT Inhibition Increases Sensitivity to Vorinostat in a ROS-Dependent Manner. Cancers.

[66] Fang K., Du S., Shen D., Xiong Z., Jiang K., Liang D., Wang J., Xu H., Hu L., Zhai X. (2022). SUFU suppresses ferroptosis sensitivity in breast cancer cells via Hippo/YAP pathway. iScience.

[67] Yang W.H., Lin C.C., Wu J., Chao P.Y., Chen K., Chen P.H., Chi J.T. (2021). The Hippo Pathway Effector YAP Promotes Ferroptosis via the E3 Ligase SKP2. Mol. Cancer Res..

[68] Mai T.T., Hamaï A., Hienzsch A., Cañeque T., Müller S., Wicinski J., Cabaud O., Leroy C., David A., Acevedo V. (2017). Salinomycin kills cancer stem cells by sequestering iron in lysosomes. Nat. Chem..

[69] Koo J.H., Guan K.-L. (2018). Interplay between YAP/TAZ and Metabolism. Cell Metab..

[70] Dai J.Z., Wang Y.J., Chen C.H., Tsai I.L., Chao Y.C., Lin C.W. (2022). YAP Dictates Mitochondrial Redox Homeostasis to Facilitate Obesity-Associated Breast Cancer Progression. Adv. Sci..

[71] Sorrentino G., Ruggeri N., Zannini A., Ingallina E., Bertolio R., Marotta C., Neri C., Cappuzzello E., Forcato M., Rosato A. (2017). Glucocorticoid receptor signalling activates YAP in breast cancer. Nat. Commun..

[72] You M., Xie Z., Zhang N., Zhang Y., Xiao D., Liu S., Zhuang W., Li L., Tao Y. (2023). Signaling pathways in cancer metabolism: Mechanisms and therapeutic targets. Signal Transduct. Target. Ther..

[73] Luo J., Li P. (2022). Context-dependent transcriptional regulations of YAP/TAZ in stem cell and differentiation. Stem Cell Res. Ther..

[74] Shen J., Cao B., Wang Y., Ma C., Zeng Z., Liu L., Li X., Tao D., Gong J., Xie D. (2018). Hippo component YAP promotes focal adhesion and tumour aggressiveness via transcriptionally activating THBS1/FAK signalling in breast cancer. J. Exp. Clin. Cancer Res..

[75] Zhao B., Ye X., Yu J., Li L., Li W., Li S., Yu J., Lin J.D., Wang C.Y., Chinnaiyan A.M. (2008). TEAD mediates YAP-dependent gene induction and growth control. Genes Dev..

[76] Lei Q.Y., Zhang H., Zhao B., Zha Z.Y., Bai F., Pei X.H., Zhao S., Xiong Y., Guan K.L. (2008). TAZ promotes cell proliferation and epithelial-mesenchymal transition and is inhibited by the hippo pathway. Mol. Cell Biol..

[77] Yu S., Cai X., Wu C., Wu L., Wang Y., Liu Y., Yu Z., Qin S., Ma F., Thiery J.P. (2015). Adhesion glycoprotein CD44 functions as an upstream regulator of a network connecting ERK, AKT and Hippo-YAP pathways in cancer progression. Oncotarget.

[78] Zhang Z., Du J., Wang S., Shao L., Jin K., Li F., Wei B., Ding W., Fu P., van Dam H. (2019). OTUB2 Promotes Cancer Metastasis via Hippo-Independent Activation of YAP and TAZ. Mol. Cell.

[79] Yu B., Su J., Shi Q., Liu Q., Ma J., Ru G., Zhang L., Zhang J., Hu X., Tang J. (2022). KMT5A-methylated SNIP1 promotes triple-negative breast cancer metastasis by activating YAP signaling. Nat. Commun..

[80] Mao M., Jia Y., Chen Y., Yang J., Xu L., Zhang X., Zhou J., Li Z., Chen C., Ju S. (2022). HJURP regulates cell proliferation and chemo-resistance via YAP1/NDRG1 transcriptional axis in triple-negative breast cancer. Cell Death Dis..

[81] Wang X., Ji C., Hu J., Deng X., Zheng W., Yu Y., Hua K., Zhou X., Fang L. (2021). Correction to: Hsa_circ_0005273 facilitates breast cancer tumorigenesis by regulating YAP1-hippo signaling pathway. J. Exp. Clin. Cancer Res..

[82] Liu J., Ye L., Li Q., Wu X., Wang B., Ouyang Y., Yuan Z., Li J., Lin C. (2018). Synaptopodin-2 suppresses metastasis of triple-negative breast cancer via inhibition of YAP/TAZ activity. J. Pathol..

[83] Tian Q., Gao H., Zhou Y., Zhu L., Yang J., Wang B., Liu P., Yang J. (2022). RICH1 inhibits breast cancer stem cell traits through activating kinases cascade of Hippo signaling by competing with Merlin for binding to Amot-p80. Cell Death Dis..

[84] Yang H., Xue M., Su P., Zhou Y., Li X., Li Z., Xia Y., Zhang C., Fu M., Zheng X. (2022). RNF31 represses cell progression and immune evasion via YAP/PD-L1 suppression in triple negative breast Cancer. J. Exp. Clin. Cancer Res..

[85] Malla R.R., Deepak K.G.K., Merchant N., Dasari V.R. (2020). Breast Tumor Microenvironment: Emerging target of therapeutic phytochemicals. Phytomedicine.

[86] Li Y.W., Xu J., Zhu G.Y., Huang Z.J., Lu Y., Li X.Q., Wang N., Zhang F.X. (2018). Apigenin suppresses the stem cell-like properties of triple-negative breast cancer cells by inhibiting YAP/TAZ activity. Cell Death Discov..

[87] Cao D., Zhu G.-Y., Lu Y., Yang A., Chen D., Huang H.-J., Peng S.-X., Chen L.-W., Li Y.-W. (2020). Luteolin suppresses epithelial-mesenchymal transition and migration of triple-negative breast cancer cells by inhibiting YAP/TAZ activity. Biomed. Pharmacother..

[88] Ding Y., Chen X., Liu C., Ge W., Wang Q., Hao X., Wang M., Chen Y., Zhang Q. (2021). Identification of a small molecule as inducer of ferroptosis and apoptosis through ubiquitination of GPX4 in triple negative breast cancer cells. J. Hematol. Oncol..

[89] Nakatani K., Maehama T., Nishio M., Otani J., Yamaguchi K., Fukumoto M., Hikasa H., Hagiwara S., Nishina H., Mak T.W. (2021). Alantolactone is a natural product that potently inhibits YAP1/TAZ through promotion of reactive oxygen species accumulation. Cancer Sci..

[90] Cao X., Li Y., Wang Y., Yu T., Zhu C., Zhang X., Guan J. (2022). Curcumin suppresses tumorigenesis by ferroptosis in breast cancer. PLoS ONE.

[91] Li R., Zhang J., Zhou Y., Gao Q., Wang R., Fu Y., Zheng L., Yu H. (2020). Transcriptome Investigation and In Vitro Verification of Curcumin-Induced HO-1 as a Feature of Ferroptosis in Breast Cancer Cells. Oxid. Med. Cell Longev..

[92] Wang L., Wang C., Tao Z., Zhao L., Zhu Z., Wu W., He Y., Chen H., Zheng B., Huang X. (2019). Curcumin derivative WZ35 inhibits tumor cell growth via ROS-YAP-JNK signaling pathway in breast cancer. J. Exp. Clin. Cancer Res..

[93] Kim Y.N., Choe S.R., Cho K.H., Cho D.Y., Kang J., Park C.G., Lee H.Y. (2017). Resveratrol suppresses breast cancer cell invasion by inactivating a RhoA/YAP signaling axis. Exp. Mol. Med..

[94] Zhen X., Choi H.S., Kim J.H., Kim S.L., Liu R., Ko Y.C., Yun B.S., Lee D.S. (2020). Caudatin Isolated from Cynanchum auriculatum Inhibits Breast Cancer Stem Cell Formation via a GR/YAP Signaling. Biomolecules.

[95] Kim C.-L., Shin Y.-S., Choi S.-H., Oh S., Kim K., Jeong H.-S., Mo J.-S. (2021). Extracts of *Perilla frutescens* var. *Acuta* (Odash.) Kudo Leaves Have Antitumor Effects on Breast Cancer Cells by Suppressing YAP Activity. Evid.-Based Complement. Alternat. Med..

[96] Ou H.L., Wu H., Ren Y.L., Si Y., Duan Z.Q., Liu X.W. (2023). Hydnocarpin inhibits malignant progression of triple negative breast cancer via CNOT4-mediated ubiquitination and degradation of YAP. Zhongguo Zhong Yao Za Zhi.

[97] Wei C., Li X. (2020). Verteporfin inhibits cell proliferation and induces apoptosis in different subtypes of breast cancer cell lines without light activation. BMC Cancer.

[98] Jiang Y., Liu Y., Zhang Z., Yang J., Ye X., Jin Q., Chen T. (2017). Verteporfin inhibits proliferation, invasion and migration of MDA-MB-231 human breast cancer cells by down-regulating the expression of Yes-associated protein. Xi Bao Yu Fen Zi Mian Yi Xue Za Zhi.

[99] Li Y., Wang S., Wei X., Zhang S., Song Z., Chen X., Zhang J. (2019). Role of inhibitor of yes-associated protein 1 in triple-negative breast cancer with taxol-based chemoresistance. Cancer Sci..

[100] Sulaiman A., McGarry S., El-Sahli S., Li L., Chambers J., Phan A., Côté M., Cron G.O., Alain T., Le Y. (2019). Co-targeting Bulk Tumor and CSCs in Clinically Translatable TNBC Patient-Derived Xenografts via Combination Nanotherapy. Mol. Cancer Ther..

[101] Gibault F., Bailly F., Corvaisier M., Coevoet M., Huet G., Melnyk P., Cotelle P. (2017). Molecular features of the YAP inhibitor verteporfin: Synthesis of hexasubstituted dipyrrins as potential inhibitors of YAP/TAZ, the downstream effectors of the hippo pathway. Chem. Med. Chem..

[102] El-Sahli S., Hua K., Sulaiman A., Chambers J., Li L., Farah E., McGarry S., Liu D., Zheng P., Lee S.H. (2021). A triple-drug nanotherapy to target breast cancer cells, cancer stem cells, and tumor vasculature. Cell Death Dis..

[103] Iliopoulos D., Hirsch H.A., Struhl K. (2011). Metformin decreases the dose of chemotherapy for prolonging tumor remission in mouse xenografts involving multiple cancer cell types. Cancer Res..

[104] Brown J.R., Chan D.K., Shank J.J., Griffith K.A., Fan H., Szulawski R., Yang K., Reynolds R.K., Johnston C., McLean K. (2020). Phase II clinical trial of metformin as a cancer stem cell-targeting agent in ovarian cancer. JCI Insight.

[105] Xu Y., Xu T., Xiong Y., Huang J. (2022). Metformin inhibits proliferation and promotes apoptosis of HER-2 positive breast cancer cells possibly through the Hippo-YAP pathway. Nan. Fang Yi Ke Da Xue Xue Bao.

[106] Xu Y., Cai H., Xiong Y., Tang L., Li L., Zhang L., Shen Y., Yang Y., Lin L., Huang J. (2023). YAP/TAZ axis was involved in the effects of metformin on breast cancer. J. Chemother..

[107] Yang J., Zhou Y., Xie S., Wang J., Li Z., Chen L., Mao M., Chen C., Huang A., Chen Y. (2021). Metformin induces Ferroptosis by inhibiting UFMylation of SLC7A11 in breast cancer. J. Exp. Clin. Cancer Res..

[108] Ma J., Fan Z., Tang Q., Xia H., Zhang T., Bi F. (2020). Aspirin attenuates YAP and β-catenin expression by promoting β-TrCP to overcome docetaxel and vinorelbine resistance in triple-negative breast cancer. Cell Death Dis..

[109] Wang Z., Yang C., Zhang H., Gao Y., Xiao M., Wang Z., Yang L., Zhang J., Ren C., Liu J. (2022). In Situ Transformable Supramolecular Nanomedicine Targeted Activating Hippo Pathway for Triple-Negative Breast Cancer Growth and Metastasis Inhibition. ACS Nano.

